# Untangling climate and water chemistry to predict changes in freshwater macrophyte distributions

**DOI:** 10.1002/ece3.3847

**Published:** 2018-02-10

**Authors:** Robin S. Sleith, John D. Wehr, Kenneth G. Karol

**Affiliations:** ^1^ Lewis B. and Dorothy Cullman Program for Molecular Systematics The New York Botanical Garden Bronx NY USA; ^2^ Department of Biological Sciences Fordham University Bronx NY USA

**Keywords:** algae, calcium, Characeae, freshwater, macrophyte, water chemistry

## Abstract

Forecasting changes in the distributions of macrophytes is essential to understanding how aquatic ecosystems will respond to climate and environmental changes. Previous work in aquatic ecosystems has used climate data at large scales and chemistry data at small scales; the consequence of using these different data types has not been evaluated. This study combines a survey of macrophyte diversity and water chemistry measurements at a large regional scale to demonstrate the feasibility and necessity of including ecological measurements, in addition to climate data, in species distribution models of aquatic macrophytes. A survey of 740 water bodies stratified across 327,000 square kilometers was conducted to document Characeae (green macroalgae) species occurrence and water chemistry data. Chemistry variables and climate data were used separately and in concert to develop species distribution models for ten species across the study area. The impacts of future environmental changes on species distributions were modeled using a range of global climate models (GCMs), representative concentration pathways (RCPs), and pollution scenarios. Models developed with chemistry variables generally gave the most accurate predictions of species distributions when compared with those using climate variables. Calcium and conductivity had the highest total relative contribution to models across all species. Habitat changes were most pronounced in scenarios with increased road salt and deicer influences, with two species predicted to increase in range by >50% and four species predicted to decrease in range by >50%. Species of Characeae have distinct habitat ranges that closely follow spatial patterns of water chemistry. Species distribution models built with climate data alone were insufficient to predict changes in distributions in the study area. The development and implementation of standardized, large‐scale water chemistry databases will aid predictions of habitat changes for aquatic ecosystems.

## 1 INTRODUCTION

Freshwater ecosystems are rich in biodiversity, economically important, and increasingly impacted by human activities (Dodds et al., 2009; Dudgeon et al., 2006; Pimentel, Zuniga, & Morrison, 2005; Vitousek, Mooney, Lubchenco, & Melillo, 1997). Aquatic macrophytes are vital components of freshwater ecosystems; they provide numerous ecosystem services (Engelhardt & Ritchie, 2001; Wilson & Carpenter, 1999). The Characeae, or stoneworts, are a group of green macroalgae found in aquatic ecosystems on all continents except Antarctica (Wood & Imahori, 1965). Species in this family are essential parts of ecosystems: They provide forage for birds, invertebrates, and fish and are important for colonizing new habitats and stabilizing sediments (Blindow, Hargeby, & Andersson, 2002; Crawford, 1979; Schneider, García, Martín‐Closas, & Chivas, 2015). In Europe, studies of Characeae richness have shown that habitat degradation and eutrophication have led to the decline of these ecologically important species (Baastrup‐Spohr, Iversen, Dahl‐Nielsen, & Sand‐Jensen, 2013; Simons & Nat, 1996). Studies have also indicated that changes in temperature will affect Characeae species (Rojo et al., 2017), but few studies have examined how changes in climate and water chemistry will impact the distribution of Characeae across the landscape (Auderset Joye & Rey‐Boissezon, 2015). A clear understanding of the distributions and species–habitat associations of aquatic macrophytes is important for predicting the responses of aquatic systems to global change.

Previous studies have found evidence of species–environment relationships within macrophyte communities (Auderset Joye & Rey‐Boissezon, 2015; Baastrup‐Spohr, Iversen, Borum, & Sand‐Jensen, 2015; Chappuis, Gacia, & Ballesteros, 2014; Escobar, Qiao, Phelps, Wagner, & Larkin, 2016; Heegaard, Birks, Gibson, Smith, & Wolfe‐Murphy, 2001; Lambert‐Servien, Clemenceau, Gabory, Douillard, & Haury, 2006; Midwood, Darwin, Ho, Rokitnicki‐Wojcik, & Grabas, 2016; Rey‐Boissezon & Auderset Joye, 2015; Torn, Kovtun‐Kante, Herkül, Martin, & Mäemets, 2015; Wood, 1952). Rey‐Boissezon and Auderset Joye (2015) used multivariate analyses with nine chemical and environmental factors across Switzerland to identify specialist and generalist species within the Characeae. Torn et al. (2015) examined the distribution of members of the Characeae in Estonia to build a predictive model based on environmental parameters, but limited data from freshwater sites restricted the model to brackish locations. Some studies of freshwater macrophytes are focused on small scales with very high‐resolution environmental data (e.g., water chemistry data: Auderset Joye & Rey‐Boissezon, 2015), while others examine distributions at larger scales but are generally restricted to courser variables, including atmospheric climate data (e.g., Escobar et al., 2016). The use of highly indirect variables such as atmospheric climate data to model aquatic organisms is a practice that may lead to biased results. To our knowledge, the effect of using these disparate data types to predict macrophytes distributions in freshwater systems has not been rigorously assessed.

Species distribution models can be used to gain insight into ecological processes over large areas and allow prediction of distributions under past and future environmental scenarios (Elith & Leathwick, 2009). Here, we use the boosted regression tree method, which incorporates statistical and machine learning techniques and has been used in a number of studies of Characeae distributions (Midwood et al., 2016; Torn et al., 2015). To optimize predictive ability, this method integrates across many simple regression tree models to arrive at a final fitted model (Elith, Leathwick, & Hastie, 2008). A framework relating environmental variables to the physiology and development of organisms is necessary to obtain ecologically relevant species distribution models. The selection of appropriate indirect, direct, and resource variables has been identified as a key step of species distribution modeling (Austin, 2007). In this study, a mix of all three variable types is used to examine the predictive capabilities and performance of macrophyte species distribution models when using indirect, direct, and resource variables.

Characeae have a rich history of study in North America, with ever‐increasing knowledge of species occurrence and ongoing systematic studies (Allen, 1888; Pérez, Hall, McCourt, & Karol, 2014; Robinson, 1906; Scribailo & Alix, 2010; Wood & Imahori, 1965). Currently, three native genera are recognized in North America: *Chara, Nitella*, and *Tolypella*. Two other genera *Lychnothamnus* and *Nitellopsis* have been reported in the past fifty years (Geis, Schumacher, Raynal, & Hyduke, 1981; Karol et al., 2017). *Nitellopsis obtusa* (starry stonewort) has been identified as an aggressive invasive species in North America, and *Lychnothamnus barbatus* has either been present and remained undetected for centuries or is a recently introduced species (Karol & Sleith, 2017; Karol et al., 2017; Sleith, Havens, Stewart, & Karol, 2015). Studies focused on the northeastern USA have reported more than 30 taxa in Characeae in the last 200 years (Robinson, 1906; Wood & Muenscher, 1954). These studies, however, have been limited in geographic extent, and when coupled with obsolete historical determinations, demonstrate a large gap in our understanding of their ecology and a need for a more complete and quantitative survey of this region. Furthermore, in general studies of macrophyte communities (including angiosperms), members of the Characeae are often determined only to genus, despite high species richness in many parts of the world. This practice is partly due to a lack of experts working on this group in North America, which makes reliable interpretations of their distribution patterns difficult to assess.

Here, we investigate distributions and species–habitat associations using a spatially stratified survey of water bodies across the northeast USA (an area of more than 327,000 km^2^), with 55 climate and 11 chemistry variables to analyze species distribution models of ten Characeae species. These models are used to address the following questions: (1) Do species of Characeae differ predictably in regard to water chemistry and climate variables? (2) What is the relative contribution of specific variables and do the key variables differ among species? (3) Are chemistry or climate variables better predictors of species distributions at a regional scale? (4) Do alterations in chemistry and climate variables lead to shifts in species distributions under environmental change scenarios?

## 2 METHODS

### 2.1 Study system

The northeastern USA, including New York and New England (CT, MA, ME, NH, RI, VT), encompasses approximately 327,000 km^2^ and contains ecosystems ranging from barrier islands to alpine tundra. The geology of the area is varied, with the Appalachian Mountain chain running southwest to northeast. The Catskills, Adirondacks, and White Mountains make up discrete mountain regions with bedrock of sedimentary, metamorphic, and igneous classes, respectively. The history of human impact on this region since European settlement is substantial: Lowland regions have been settled and developed for centuries, with agriculture and urban land the predominant land use types. The region contains large areas of forested and protected land, but these areas also contain roads and isolated settlements, especially surrounding water bodies.

### 2.2 Species occurrence data

Characeae habitats include large permanent lakes, slow‐moving rivers, small ponds and wetlands, and temporary ditches and pools. Occurrence data were collected following the methods outlined in Sleith et al. (Sleith et al., 2015). Briefly, a stratified survey design was used to place 750 points throughout the study region; water bodies nearest each point were sampled once at a single access point from June to September in 2014 (390 sites, 10 inaccessible) and June to September in 2015 (350 sites), for a total of 740 surveyed sites. Samples were collected using a combination of wading and tossing a dredge; as much as was possible, equal sampling intensity occurred at each site, with a target time of 30 min of searching per site. Species were identified primarily using diagnostic morphology; specimens that lacked clear characters (often sterile specimens) were identified using the plastid‐encoded gene *psbC* (Pérez et al., 2014). Species with fewer than 12 records were excluded from further analysis. Voucher specimens were deposited in The William and Lynda Steere Herbarium (NY) and when possible, duplicates were distributed to the Academy of Natural Sciences of Drexel University (PH) and the Norton‐Brown Herbarium, University of Maryland‐College Park (MARY). Taxonomic nomenclature follows Algaebase (Guiry & Guiry 2018).

### 2.3 Environmental data

#### 2.3.1 Water chemistry data

Field water chemistry data were collected with an In Situ SmarTroll MP (Ft. Collins, CO, USA) as outlined in Sleith et al. (Sleith et al., 2015). Water samples for dissolved nutrient and metal concentrations were syringe‐filtered (0.2 μm pore size) and acid preserved to pH < 2.0 (USEPA, 1987) and stored at 4°C, until analysis at The Louis Calder Center of Fordham University (details, Table 1). Eighteen sites with a chemistry or nutrient value seven times the standard deviation above the mean were labeled as outliers and removed from subsequent analyses. Removal of outliers resulted in 722 sites from which rasters were built. Inverse distance weighting interpolation of each chemical parameter was performed using the “gstat” package (Pebesma, 2004) in R (R Core Team, 2015), with nmax = 4 and idp = 0.5. The interpolation function of the “raster” package (Hijmans, 2015) was then used to create rasters with a cell size of 30 s (of a longitude/latitude degree) from the gstat results.

**Table 1:** Chemical variables used to build species distribution models, with analysis technique, variable type after (Austin, 2007), and summed relative contributions from boosted regression tree model outputs

Parameter	Technique	Units	Min	Mean	Max	Variable type	Summed relative contribution
Calcium	Atomic absorption	mg/L	0.3	24	350	Resource	180
Conductivity	In situ Smartroll MP	μS/cm	10.0	204	28,427	Direct	138
Dissolved organic carbon (DOC)	TOC analyzer	mg/L	2.0	9	123	Direct	109
Dissolved oxygen	In situ Smartroll MP	mg/L	0.3	8	17	Direct	57
Magnesium	Atomic absorption	mg/L	0.1	4	46	Resource	105
Nitrogen as ammonium (NH_4_^+^)	Astoria‐pacific analyzer	μg/L	2.0	50	937	Resource	49
Nitrogen as nitrate (${\text{NO}}_{3}^{-}$)	Astoria‐pacific analyzer	μg/L	0.1	120	7,190	Resource	85
Oxidation reduction potential	In situ Smartroll MP	mV	−93	123	453	Direct	70
pH	In situ Smartroll MP	–	4.85	7.62	10.13	Direct	80
Phosphorus as soluble reactive phosphate (SRP)	Astoria‐pacific analyzer	μg/L	0.3	9	1,217	Resource	52
Phosphorus as total dissolved phosphate (TDP)	Astoria‐pacific analyzer	μg/L	2.0	21	2,609	Resource	73

#### 2.3.2 Climate data

Current climate data (1960–1990) were obtained at 30 s (about 900 m at the equator) resolution from WorldClim 1.4 (Hijmans, Cameron, Parra, Jones, & Jarvis, 2005). The WorldClim dataset includes monthly total precipitation, monthly minimum and maximum temperature, as well as 19 derived bioclimatic variables. The fifth phase of the Coupled Model Intercomparison Project (CMIP5) produced 20 global climate models (GCM) that conform to the emissions scenarios from the IPCC fifth assessment (IPCC, 2014). These models are downscaled and calibrated using the WorldClim 1.4 current climate data (Hijmans et al., 2005) and allow projections of species distributions under future climate scenarios. Five models, produced by a range of governments from across the world, were selected that were complete for all years and emissions scenarios (CCSM4, GISS‐E2‐R, HadGEM2‐AO, MIROC5, NorESM1‐M). Climate data were modified to match the extent of chemistry rasters using the mask function of the “raster” package (Hijmans, 2015). One‐way ANOVA of predicted habitat area was used to determine the sources of variance in predicting future habitats under GCM scenarios.

#### 2.3.3 Future environmental scenarios

Climate change in the northeast USA is predicted to increase winter precipitation and, concurrently, the use of road salts and deicers (Hayhoe et al., 2008). Increased use of road salt and deicers has been linked to direct increases in conductivity and cation concentrations of water bodies (Dugan et al., 2017), and indirect leaching of calcium and magnesium ions into surface waters through increased competition in the cation exchange complex. To simulate increased road salt and deicer use leading to increased calcium, conductivity, and magnesium, these values were increased incrementally. To capture changes occurring with only a small increase, values were increased by 0.01 of the standard deviation of each variable, for ten steps. Changes occurring under more substantial increases were achieved by an increase of 0.1 of the standard deviation for ten additional steps, leading to a range of 20 increase steps from 0.01 to 1 standard deviation.

Runoff from agricultural and human development has been identified as the major source of nitrogen and phosphorus in freshwater ecosystems (Carpenter et al., 1998), and many of the sample sites were located in agricultural areas. To simulate increased runoff of agricultural fertilizers and leaching wastewater, nitrogen in the forms of ammonium (NH^4+^) and nitrate (${\text{NO}}_{3}^{-}$), and phosphorus as soluble reactive phosphate (SRP), and total dissolved phosphorus (TDP) were also simulated as increased using the standard deviation method described for cations.

### 2.4 Species distribution modeling

Boosted regression tree models were built and evaluated using three sets of predictor variables: all chemistry variables (11), all climate variables (55), and all variables combined (66). Occurrence data were split into equal training and testing sets. Models were built using the gbm.step function of the “dismo” package (Hijmans, Phillips, Leathwick, & Elith, 2016) with a range of tree complexities (3, 5, 7, 10, 20), learning rates (0.01, 0.005, 0.001), and bagging fractions (0.5, 0.75, 0.9). Models were then evaluated using the predict.gbm function, and the model settings with the lowest deviance and highest area under the receiver operating characteristic curve (AUC) were selected (Elith et al., 2008). Final models were run for 100 iterations with all data and evaluated using 10‐fold cross validation (Elith et al., 2008).

The predict function of the “raster” package was used to predict model results onto raster objects for subsequent analysis of species distributions. To quantify the influence of threshold selection, predictions of suitable habitat were made based on three thresholds, two calculated by the evaluate and threshold functions of the “dismo” package in R (no_omission, equal_sens_spec) and the cross‐validated threshold produced by gbm.step (cv.threshold). Suitable habitat above a given threshold was calculated using the calc and cellStats functions in “raster” (Hijmans, 2015).

## 3 RESULTS

Water chemistry values varied widely across the study region (Table 1). In particular, dissolved Ca, ${\text{NO}}_{3}^{-}$, and TDP varied by >1,000‐fold, and conductivity, NH_4_^+^, and SRP by >100‐fold, among sampling sites. Interpolated rasters of these chemical data were consistent with geologic and land use maps, leading to distinct regional patterns of water chemistry (e.g., large areas of soft water in the Adirondack, Catskill, and Northern Appalachian mountain regions, Figure S3). Twenty‐seven Characeae species were collected from 363 of the 740 surveyed small wetlands, ponds, rivers, lakes, and nearshore regions of Lake Ontario. Ten species were selected for building species distribution models, with prevalence ranging from 13 to 147 water bodies in the study region (Table 2). At least one “fair performance” model (AUC > 0.7) could be constructed for all species (Table 2). The highest mean AUC across the ten Characeae species was achieved with models that combined climate and chemistry variables (mean AUC ± *SD*: chemistry = 0.808 ± 0.087; climate = 0.750 ± 0.083; combined = 0.823 ± 0.074); however, the best performing climate or chemistry model for a given species was improved by no more than 0.05 AUC units. For this reason, all forecasting scenarios were analyzed separately for climate and chemical models.

**Table 2:** Ten species of Characeae modeled in this study, with number occurrences from the 363 sites with Characeae. Boosted regression tree area under the curve (AUC) scores for models constructed with chemistry data, climate data, and combined chemistry and climate data. Percent habitat change shown for two environmental change scenarios, with three calculated thresholds (cv = cross validated, eq = equal sensitivity specificity, no = no omission) showing variation of predicted habitat. All values are means of 100 iterations

Speies	Occurrences	Chemistry AUC	Climate AUC	Combined AUC	% Habitat change cations 0.4 *SD* cv/eq/no	% Habitat change nutrients 0.4 *SD* cv/eq/no
*Chara contraria*	37	0.931	0.764	0.919	+164/+102/+83	−98/−58/−32
*Chara vulgaris*	13	0.885	0.720	0.894	+51/+36/+8	+323/+219/+159
*Nitella* aff. *flexilis*	49	0.767	0.786	0.783	−100/−96/−47	−38/−17/−5
*Nitella* aff. *tenuissima*	39	0.766	0.734	0.774	−93/−79/−54	−100/−100/−60
*Nitella flexilis*	147	0.723	0.611	0.744	−25/−19/−5	+52/+43/+1
*Nitella furcata*	34	0.777	0.656	0.747	−66/−59/−17	−46/−31/−18
*Nitella* sp.	18	0.781	0.862	0.836	−42/−57/−50	−91/−15/+16
*Nitella microcarpa*	22	0.830	0.752	0.836	−100/−100/−100	+7/+3/−2
*Nitella transilis*	19	0.680	0.736	0.759	−100/−88/−64	−88/−8/+14
*Nitellopsis obtusa*	27	0.943	0.883	0.941	+67/+67/+65	+51/+42/+37

**Figure 1:**
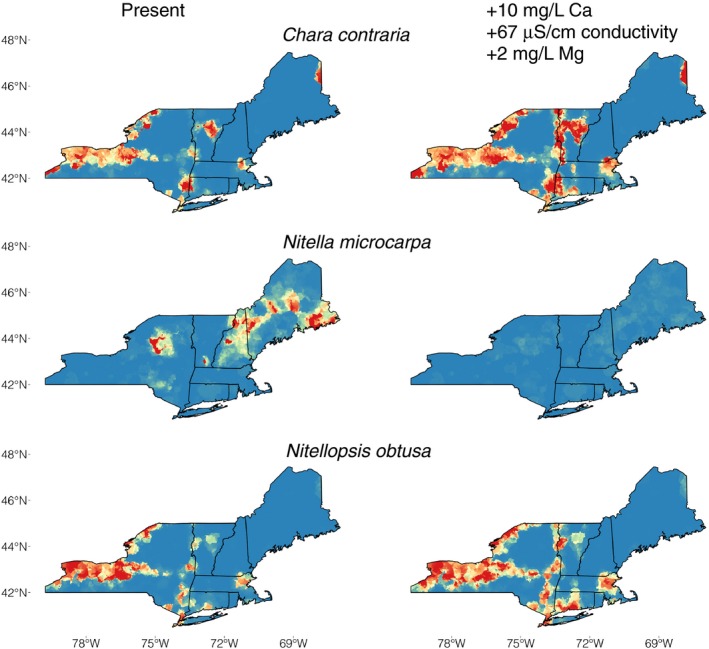
Boosted regression tree models for the three species of Characeae across NY, VT, NH, ME, MA, RI, CT, USA (clockwise from left). Models on left are present day, models on right are predictions for an increase of +10 mg/L Ca, +67 μS/cm conductivity, and +2 mg/L Mg. Color gradient of suitability ranges from low (blue) to high (red) predicted habitat suitability

**Figure 2:**
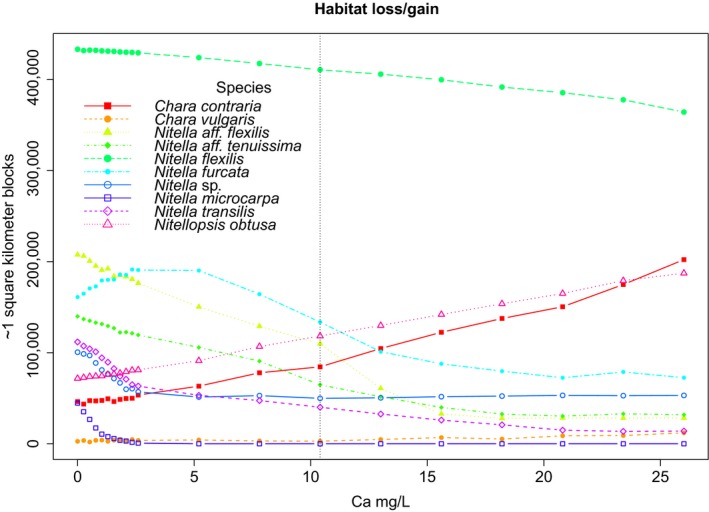
Changes in predicted habitat for all species under increased conductivity and concentrations of dissolved calcium and magnesium. First ten steps are increased by 0.01 standard deviation; the next ten steps are increased by 0.1 standard deviation. The variable with the highest relative contribution, calcium, is shown in the *x*‐axis. The vertical dotted line indicates the increase scenario chosen for use in Figure 1 and Table 2

Models built with chemical variables outperformed models built with climate variables in seven of 10 species (Table 2). Calcium, conductivity, and dissolved organic carbon had the highest total relative contribution across all species (Table 1). Area of predicted suitable habitat was highly dependent on threshold choice; no omission was consistently the most conservative threshold (Table 2). Increases of calcium, conductivity, and magnesium led to substantial habitat changes for six species regardless of threshold selection (all thresholds ≥50% ± 5% change), with two species predicted to increase in range by >50% and four species predicted to decrease in range by >50% under a scenario of increases of +10 mg/L Ca, +67 μS/cm conductivity, and +2 mg/L Mg (Figures 1, 2, S1 and S2). The remaining four species models showed changes that were either less severe or had high disagreement between thresholds. Predicted increases in dissolved nutrients, including NH_4_^+^, ${\text{NO}}_{3}^{-}$, SRP, and TDP, led to less extreme habitat changes and greater variability between predicted habitat and threshold selection (Table 2).

**Figure 3:**
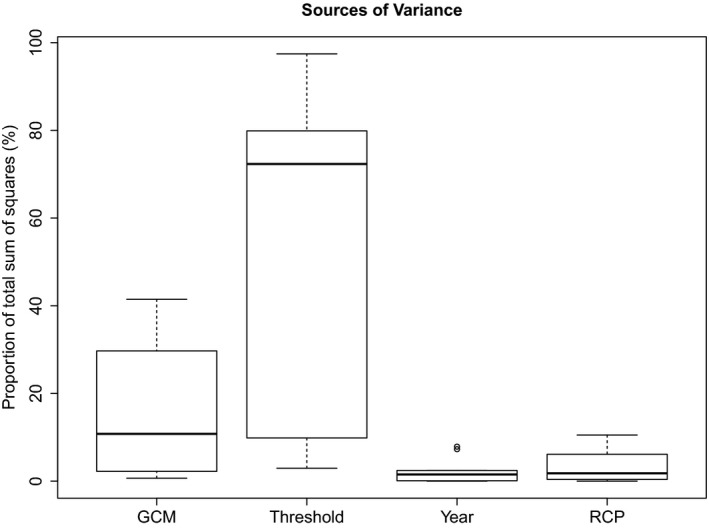
Boxplots showing the proportion of the total sum of squares from one‐way ANOVA for GCM, threshold, year, and representative concentration pathway for habitat area predictions for the ten species studied. The box boundaries show the interquartile range; whiskers identify data points that are no more than 1.5 times the interquartile range

Climate values also varied widely across the region: The average monthly maximum temperature in July ranged from 13.1 to 30°C, while the average monthly precipitation in July ranged from 61 to 176 mm. Models built with climate variables outperformed models built with chemical variables for three of the 10 species examined. Mean temperature of the wettest quarter (bio8), mean diurnal range (bio2), and average precipitation in September (prec9) had the highest total relative contribution across all species (Tables S1 and S2). Changes in predicted habitat under CMIP5 climate scenarios were highly variable, with threshold and global climate model contributing the highest proportion of variance for all species (Table 2, Figure 3).

## 4 DISCUSSION

The varied topography, geologic history, and land use of the northeastern USA make this region a valuable system to understand the distribution of Characeae species in North America. Some Characeae species are known to be dispersed by waterfowl (Proctor, 1962, 1968), which hypothetically allows for near‐complete dispersal across the northeastern USA since the last glacial maximum. This study found that species of Characeae occur in a variety of aquatic habitats including small wetlands, ponds, rivers, lakes, and nearshore regions of Lake Ontario, but do so in ways that are predictable using key ecological variables. The species occurrence data also indicate that predictive models of Characeae distribution are best made across the landscape and not restricted to individual water bodies. The goal of this study was to examine how chemistry and climate data perform when modeling Characeae distributions. Other variables, such as water body size and local land use, may play a role in these distributions but would hinder the explanatory ability of the models; therefore, these variables were not included in this study.

The distributions of Characeae species were accurately modeled with water chemistry variables (Table 2). For example, specialist species (i.e., *Chara contraria* and *Nitella microcarpa*) were very well supported and had single variables with high relative contribution, although more generalist species such as *N. flexilis* were less well supported and did not have a single predictor variable with a high relative contribution (Table 2). The distribution of specialist species appears to follow a pattern of equal dispersal across the landscape, with differential success based on environmental conditions (Figure 1). For example, *C. contraria* was not detected across thousands of square kilometers in interior portions of Maine, where softer water occurs, but was detected in the far eastern portions of the state, where limestone deposits promote hard water conditions. It is likely that *C. contraria* has been dispersed into portions of interior Maine but has not established due to unsuitable chemical conditions. Controlled field or mesocosm experiments are necessary to determine the tolerance of these species to a wider range of chemical conditions.

Calcium was identified as having the greatest relative contribution to distributions of most species of Characeae in the present study across the northeastern USA. In Europe, variation in calcium concentrations has been identified as discriminating among Characeae habitats, with calcifuge and calciphile species being described (Auderset Joye & Rey‐Boissezon, 2015; Olsen, 1944; Rey‐Boissezon & Auderset Joye, 2015; Stroede, 1933). Calcium can be regarded as a resource variable as it is a required nutrient; however, this element also serves as a direct variable in models, as an indicator of bedrock substrate and water hardness. Sources of calcium in water bodies include weathering of bedrock, leaching from catchment soils, and runoff from human activities.

Projections of the effects of future environmental change found that some species may increase their range and others will decrease, in agreement with other studies of Characeae (Auderset Joye & Rey‐Boissezon, 2015). Although pollutants are likely to be unevenly distributed across the landscape, and road density varies across the study area, aquatic ecosystems are nevertheless impacted by human activities that may originate far from a given water body. Lakes and ponds in the more remote regions of the present study area are accessible by roads and many are highly developed with part‐ or full‐time residences. For this reason, and as an initial estimate, concentrations of cations and nutrients were increased evenly in predictive models across the study region. To calculate and compare areas of suitable habitat, a threshold must be selected; determination of an appropriate threshold has been the subject of much debate in the species distribution literature (Nenzén & Araújo, 2011). Our results show that the magnitude of predicted habitat loss or gain was influenced by choice of threshold and that the no omission threshold predicts the most conservative estimate of future distributions.

Characeae species appear to be particularly sensitive to changes in cation concentrations and conductivity. For example, *Nitella microcarpa* is predicted to lose all suitable habitat, given increases of +5 mg Ca/L, +34 μS/cm conductivity, and +1 mg Mg/L. Species distribution models are limited in their ability to discover causal links between the environment and species traits (Elith & Leathwick, 2009). Experiments are therefore needed to determine the exact mechanism of toxicity and tolerance for these sensitive species. In our study area *N. microcarpa* occurs in mountainous regions where predicted increases of winter precipitation (Hayhoe et al., 2008) will likely lead to increased use of road salts and deicers. The New York State Department of Transportation annually applies 728,750 metric tons of salt, 160,047 L of calcium chloride, and 586, 227 L of magnesium chloride to highways (NYDOT). The influence of road salts and dicers on the cation concentrations of lakes and ponds in the Adirondack Park of New York (an area of highly suitable habitat for *N. microcarpa*) has been demonstrated, with road density explaining 84% of the variation in ion concentrations (Kelting, Laxson, & Yerger, 2012). Changes in the deicing formulation and continued enforcement of existing “reduced salt areas” may help protect this and other sensitive species.

In contrast, the invasive species *Nitellopsis obtusa* was predicted to increase in its range by over 65% under the same increased cation scenario. Predicting distributions of invasive species is nonetheless problematic (Jiménez‐Valverde et al., 2011). These species are rarely at equilibrium in their environment, and for an invasive species like *Nitellopsis obtusa,* which is primarily dispersed via human activity, the estimation of habitat preference is difficult to disentangle from chance dispersal events. However, predictive models built for invasive species, if interpreted with a full understanding of potential limitations, can be viewed as a conservative estimate of the species potential distribution. Furthermore, *Nitellopsis obtusa* co‐occurs at eight of 27 sites with *Chara contraria,* a hard water specialist that is also predicted to increase in its range under increased cation scenarios. These results therefore predict that the Mohawk, Hudson, and Lake Champlain regions are likely to have more suitable habitat for *Nitellopsis obtusa* (Figure 1).

Predicted habitat changes from agricultural and wastewater runoff were less pronounced: These variables had low relative contributions to models and therefore are not expected to influence distributions as strongly (Table 2). However, increases in P and N will increase overall productivity of aquatic ecosystems and may adversely affect Characeae species through decreased transparency (light limitation) and/or increased competition. Previous work has established that Characeae species are scarce in eutrophic water bodies and that this scarcity is related to light availability (Blindow, 1992).

Models built with climate variables generally performed less effectively than chemistry‐based models (Table 2). The boosted regression tree technique is well suited to the large number of predictor variables used in this study, as it is able to ignore noninformative predictors, and boosting has been found to generate robust models even when variables are collinear (Maloney, Schmid, & Weller, 2012). Climate change projections were variable when five GCMs, four representative concentration pathways (RCP), and two time points (2050 and 2070) were modeled. Across all species, choice of GCM and threshold contributed more variance to habitat projection than RCP or time point. The northeastern USA has one of the steepest climate gradients in the country (Hayhoe et al., 2008), and predicting changes across this region has many challenges. The most influential variable, mean temperature of the wettest quarter (bio8), is strongly influenced by the Atlantic Ocean coastline and Lake Ontario, with variable GCM predictions due to these factors. Furthermore, when modeling aquatic organisms, atmospheric climate variables are largely indirect; this leads to models built with tenuous correlations to the biological processes that determine species distributions (Wiens, Stralberg, Jongsomjit, Howell, & Snyder, 2009). Models may be able to accurately capture species distributions under current conditions, but projections of future distributions break down due to weakly correlated associations with biological processes. In a study of Characeae distributions in Switzerland, Auderset Joye and Rey‐Boissezon (2015) found that mean July temperature was a strongly contributing factor explaining Characeae distributions. Expansion of these species distribution models to different geographic areas at different scales will aid in determining the relationship of climate variables to Characeae distributions. The incorporation of new variables, such as those in development for WorldClim 2 (Fick & Hijmans, 2017), may also improve the ability of global climate data to predict changes in freshwater ecosystems.

Freshwater ecosystems are increasingly threatened, and macrophyte communities play an important role in the functioning of these ecosystems. The utility and accuracy of species distribution models depend heavily on knowledge of the organism and study system, appropriate selection of environmental data, and pragmatic model tuning and refinement (Austin, 2007; Elith & Leathwick, 2009). In aquatic systems, the set of variables that influence the occurrence of a species may be hierarchical and may differ at various scales (Poff, 1997). At the continental scale, climate may determine broad patterns of distribution, while at a regional and local scale, entirely different variables, including water chemistry, may be responsible for species occurrence patterns. This study demonstrates that a boosted regression tree method was able to accurately predict Characeae distributions over a large area and that this method was especially useful because it ignored noninformative predictors and provided a quantitative understanding of the influence of all key variables on the patterns of their distributions (Elith et al., 2008). This study shows that different species of Characeae have distinct geographic ranges that are responsive to chemical and climatic conditions. At the scale of northeastern USA, models constructed with chemical variables performed better than models built with climate variables. In addition, models constructed with chemical variables led to consistent future projections under environmental change scenarios. This work has demonstrated the advantage of incorporating water chemistry measurements into species distribution models of aquatic organisms. The development of standardized, continental‐scale water chemistry datasets will require cooperation across academic and political boundaries and will lead to a better ability to study and conserve valuable freshwater habitats.

## CONFLICT OF INTEREST

None declared.

## AUTHOR CONTRIBUTIONS

RSS, JDW, and KGK conceived the study concepts. RSS collected the data. RSS, JDW, and KGK analyzed and interpreted the data. RSS led the writing of the manuscript, with input from JDW and KGK. All authors approved the final manuscript.
