# Supplementary material for: Untangling climate and water chemistry to predict changes in freshwater macrophyte distributions
Source: Ecol Evol. 2018 Feb 10;8(5):2802–11. doi: 10.1002/ece3.3847 (PMC5838067; doi:10.1002/ece3.3847)
Supplement: Supplementary file 1 [file ECE3-8-2802-s001.docx]

| **Species** | **Highest relative contribution variable for chemistry model** | **Highest relative contribution variable for climate model** | **GCM** | **Threshold** | **RCP** | **Year** |
| --- | --- | --- | --- | --- | --- | --- |
| ***Chara contraria*** | Ca (44) | prec7 (10) | 16*** | 72*** | 0.4 | 0.1 |
| ***Chara vulgaris*** | Mg (21) | bio19 (22) | 41*** | 11*** | 6*** | 8*** |
| ***Nitella* aff. *flexilis*** | Conductivity (27) | bio8 (21) | 2* | 79*** | 2** | 1** |
| ***Nitella* aff. *tenuissima*** | DOC (16) | bio2 (14) | 1*** | 97*** | 0.01 | 0.02 |
| ***Nitella flexilis*** | Ca (19) | bio7 (7) | 5*** | 73*** | 10*** | 2*** |
| ***Nitella furcata*** | Ca (18) | prec8 (7) | 6*** | 81*** | 0.08 | 0.001 |
| ***Nitella* MA01** | Nitrate (30) | prec9 (7) | 18*** | 6* | 2 | 2 |
| ***Nitella microcarpa*** | Conductivity (38) | bio8 (11) | 30*** | 3* | 11*** | 7*** |
| ***Nitella transilis*** | DOC (22) | bio17 (14) | 2*** | 80*** | 2*** | 6*** |
| ***Nitellopsis obtusa*** | Ca (25) | prec5 (11) | 37*** | 10*** | 0.5 | 0.2 |

Supplementary Table 1. Relative contributions for boosted regression tree models for each species for chemistry and climate variables (climate variables correspond to WorldClim 1.4 naming conventions). The proportion of the total sum of squares from one-way ANOVA for GCM, threshold, year, and representative concentration pathway (RCP) for habitat area predictions for the ten species studied are shown, with significances noted as <0.001 ***, 0.001**, 0.01*.

| **Variable** | **Description** | **Summed Relative Contribution** |
| --- | --- | --- |
| **bio8** | Mean Temperature of Wettest Quarter | 75.13802042 |
| **bio2** | Mean Diurnal Range | 47.27843489 |
| **prec9** | Total Precipitation in September | 39.00589749 |
| **prec6** | Total Precipitation in June | 37.99704348 |
| **bio9** | Mean Temperature of Driest Quarter | 35.44328336 |
| **bio19** | Precipitation of Coldest Quarter | 33.23793009 |
| **bio4** | Temperature Seasonality | 31.49357971 |
| **prec7** | Total Precipitation in July | 31.33352751 |
| **bio16** | Precipitation of Wettest Quarter | 31.33300097 |
| **bio17** | Precipitation of Driest Quarter | 31.29895311 |

Supplementary Table 2. Summed relative contribution of the ten WorldClim climate variables with the highest summed relative contribution.

Supplementary Figure 1. Boosted regression tree models for four species of Characeae across CT, MA, ME, NH, NY, RI, VT U.S.A. Models on left are present day, models on right are predictions for an increase of +10 mg/L Ca, +67 μS/cm conductivity, and +2 mg/L Mg. Color gradient of suitability ranges from low (blue) to high (red) predicted habitat suitability.

Supplementary Figure 2. Boosted regression tree models for three species of Characeae across CT, MA, ME, NH, NY, RI, VT U.S.A. Models on left are present day, models on right are predictions for an increase of +10 mg/L Ca, +67 μS/cm conductivity, and +2 mg/L Mg. Color gradient of suitability ranges from low (blue) to high (red) predicted habitat suitability.


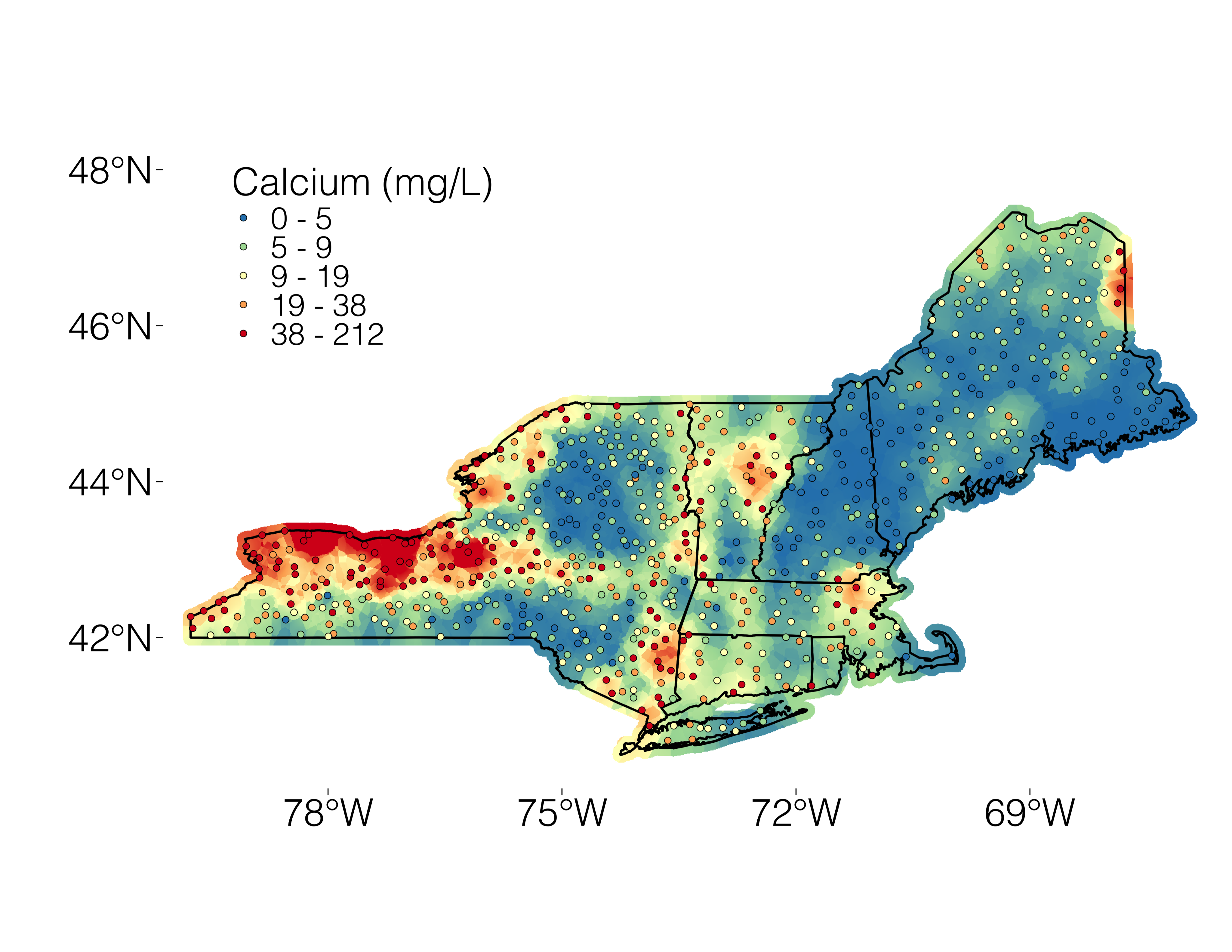


Suplementary Figure 3. The 722 collection localities used in this study, with calcium (mg/L) concentrations and interpolated raster of calcium values.
